# Feasibility of elective neck irradiation in treating node-negative nasopharyngeal carcinoma patients: a meta-analysis

**DOI:** 10.3389/fonc.2025.1456724

**Published:** 2025-05-22

**Authors:** Jianfeng Zhou, Hongyong Wang

**Affiliations:** ^1^ Shenzhen Hospital, Southern Medical University, Shenzhen, China; ^2^ Second Affiliated Hospital of Jilin University, Changchun, China

**Keywords:** intensity modulated radiation therapy, nasopharyngeal carcinoma, node-negative, whole neck irradiation, upper neck irradiation

## Abstract

**Purpose:**

Intensity modulated radiation therapy (IMRT) has replaced conventional two-dimensional radiation therapy as the mainstream radiation therapy for nasopharyngeal carcinoma. However, side effects continue to be a significant concern during the radiotherapy process for nasopharyngeal carcinoma patients. The recognized target area for cervical prophylaxis in nasopharyngeal carcinoma is whole neck irradiation (WNI), Currently, some studies have confirmed that upper neck irradiation (UNI) may be feasible as a preventive measure for NPC patients with negative neck lymph nodes. This meta-analysis aimed to comprehensively investigate and compare the efficacy of selective UNI and WNI in improving survival outcomes and regional control in patients with lymph node-negative NPC.

**Materials and methods:**

A systematic search was conducted in PubMed, EMBASE, The Cochrane Library, and CNKI from inception to October 27, 2023, using a combination of Medical Subject Headings (MeSH) terms and free-text keywords related to “nasopharyngeal carcinoma,” “lymph node negative,” and “neck irradiation.” The titles and abstracts of the retrieved articles were reviewed.

**Results:**

Our meta-analysis of 11 studies revealed no significant differences in overall survival (OS), disease-free survival (DFS), distant metastasis-free survival (DMFS), or lymph node recurrence between the UNI and WNI groups for patients with cervical lymph node-negative nasopharyngeal carcinoma. These findings suggest that UNI is a safe and feasible treatment option for this patient population.

**Conclusion:**

UNI is a safe and feasible option for nasopharyngeal carcinoma patients with negative cervical lymph nodes or only retropharyngeal lymph node (RLN) metastases. UNI may reduce the radiation dose to normal tissues, potentially decreasing long-term adverse effects and improving quality of life.

## 1 Introduction

Intensity-modulated radiation therapy (IMRT) has supplanted conventional two-dimensional radiation therapy as the mainstream treatment for nasopharyngeal carcinoma (NPC). However, side effects remain a significant concern during radiotherapy for NPC patients. For example, radiation-induced skin reactions are notable adverse effects that need to be addressed (1). NPC is characterized by a rich lymphatic network, with a predictable pattern of lymph node metastasis that typically progresses sequentially along the neck and rarely exhibits skip metastasis (2). The most commonly involved regions include RLN and levels II, III, IV, and VA lymph node regions. Additionally, involvement can occur in the IB region and the supraclavicular lymph nodes (3). According to the latest cervical lymph node delineation standards from the European Society of Radiotherapy & Oncology (ESTRO) published in 2013, the supraclavicular lymph nodes are defined as regions VB and VC (4). A meta-analysis of 2,920 NPC patients staged using MRI revealed that the most common regions of lymph node metastasis were RLN at 69%, and level II at 70%. This is followed by level III at 45%, level IV at 11%, and level VA at 27%. The involvement rates for the supraclavicular lymph nodes, level IA, level IB, level VI, and parotid lymph nodes are 3%, 0%, 3%, 0%, and 1%, respectively (5). These data raise the question: is it necessary to include the entire neck in the radiation field, regardless of the lymph node status of NPC patients? Currently, whole neck irradiation (WNI) is universally accepted as the standard prophylactic target for neck radiation in patients with NPC, irrespective of the presence of lymph node metastasis in the neck (6). Given this perspective, it is worth reconsidering whether it is necessary to include the entire neck in the prophylactic target area for radiation therapy in lymph node-negative NPC patients. Recent studies have suggested that irradiating the upper neck (UNI), specifically targeting levels II, III, and VA, may be feasible as part of neck prophylactic radiation in lymph node-negative NPC patients. Ma et al. (7) conducted an open-label, noninferiority, randomized phase III trial between January 22, 2016, and May 23, 2018, enrolling 446 patients with N0-N1 nonmetastatic nasopharyngeal carcinoma. They found that selective UNI for the noninvolved neck region provided similar regional control compared to standard WNI, with reduced radiation toxicity. A recent meta-analysis studying the efficacy and radiation-related toxicity of UNI versus WNI in patients with unilateral or bilateral lymph node-negative nasopharyngeal carcinoma showed that UNI offers comparable efficacy and less toxicity than WNI (8). Selective UNI, which preserves uninvolved neck regions, appears to be an effective option for treating N0, N1, or even unilateral N3 disease in patients with NPC. However, further clinical data are needed to substantiate this approach. Zeng et al. (9) conducted a retrospective analysis from January 2003 to October 2008 involving 270 patients with lymph node-negative NPC. Among these patients, 171 received selective UNI targeting levels II, III, and VA, while 99 patients received WNI. The median follow-up period was 65.1 months (range 4–106 months).

The 5-year overall survival (OS), nodal recurrence-free survival (NRFS), and distant metastasis-free survival (DMFS) rates were 93.6% and 90.9% (p=0.553), 99.4% and 99.0% (p=0.278), and 98.8% and 94.9% (p=0.128) for the UNI and WNI groups, respectively, showing no significant differences. Additionally, UNI was associated with reduced radiation-related side effects, potentially improving patients’ quality of life.

This meta-analysis aimed to comprehensively investigate and compare the efficacy of selective UNI and WNI in improving survival outcomes and regional control in patients with lymph node-negative NPC.

## 2 Materials and methods

A systematic search was conducted in PubMed, EMBASE, The Cochrane Library, and CNKI from inception to October 27, 2023, using a combination of Medical Subject Headings (MeSH) terms and free-text keywords related to “nasopharyngeal carcinoma,” “lymph node negative,” and “neck irradiation.” The titles and abstracts of the retrieved articles were reviewed.

### 2.1 Inclusion and exclusion criteria

The inclusion criteria were as follows: 1) All patients had histologically confirmed NPC. 2) Clinical examinations (palpation, imaging, pathology) revealed no lymph node metastasis in the neck or only RLN involvement. 3) Studies comparing the efficacy of selective UNI and WNI as prophylactic neck radiation. 4) Studies providing prognosis outcomes and survival data, such as overall survival (OS), disease-free survival (DFS), distant metastasis-free survival (DMFS), and lymph node recurrence. 5) Literature with clearly defined follow-up endpoint data or survival curves.

Exclusion Criteria: 1) Studies lacking a control group. 2) Studies without clearly defined follow-up endpoint data or distinct survival curves. 3) Original studies with inadequate experimental design or inappropriate statistical methods.

### 2.2 Literature screening and data extraction

The titles and abstracts from the selected databases were initially screened based on the search criteria. After removing duplicates, a two-round screening process was conducted according to the inclusion and exclusion criteria. The first round involved screening based on titles and abstracts, while the second round involved a full-text review. Data extraction was performed using a structured form. The extracted information included the title of the literature, first author, journal and year of publication, sample size, age distribution, intervention methods (UNI or WNI), details of radiation therapy techniques and doses, prognosis outcomes, and other relevant data.

### 2.3 Quality assessment of studies

For randomized controlled trials (RCTs), the modified Jadad scale (10) was used to assess the quality of the literature. Studies scoring 1–3 points were considered low-quality, while those scoring 4–7 points were considered high-quality. For retrospective studies, the Newcastle-Ottawa Scale (NOS) (11) was used. Studies scoring 1–4 points were considered low quality, those scoring 5–7 points were considered moderate quality, and those scoring 8–9 points were considered high quality.

### 2.4 Statistical analysis

According to the Cochrane Handbook, statistical data analysis was conducted using RevMan 5.4 software. The results are presented as forest plots. The study analyzed categorical variables with 95% confidence intervals (CIs). For count data, odds ratios (ORs) were calculated. Heterogeneity was assessed using the I² statistic: I² ≤ 50% indicated no significant heterogeneity and was analyzed using a fixed-effects model; I² > 50% indicated significant heterogeneity and was analyzed using a random-effects model. If more than 10 studies were included, a funnel plot was generated to assess publication bias. A significance level of p < 0.05 indicated a statistically significant difference.

## 3 Results

### 3.1 Search results

**Figure 1:**
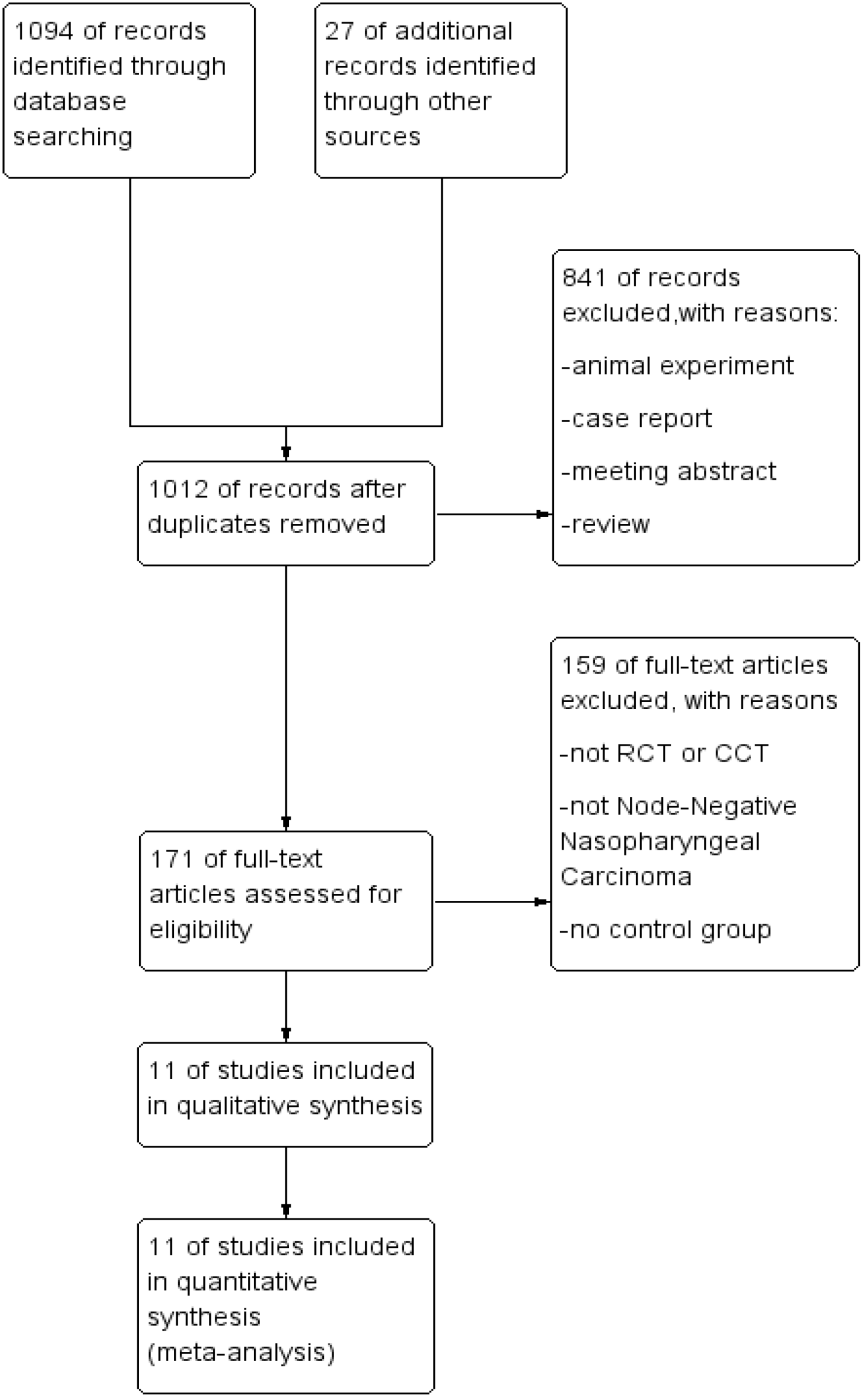
Literature screening process.

A total of 1,094 relevant articles were retrieved from PubMed, EMBASE, The Cochrane Library, and CNKI. After an initial review of titles and abstracts, followed by a full-text evaluation based on the inclusion and exclusion criteria, 11 articles were identified as meeting the criteria for inclusion. The screening process is detailed in **Figure 1**.

**Table 1** presents the baseline characteristics of the 11 included studies. Among these, 10 were retrospective studies (2, 9, 12–14, 16–20), and one was a randomized controlled trial (RCT) (15). Seven studies were published in English (2, 9, 12, 15–18), while four were published in Chinese with English abstracts (13, 14, 19, 20).

**Table 1:** Baseline characteristics of the 11 included studies.

Author(s) and Year	Study Type	Time Period	Patient Number (UNI/WNI)	Lymph Node Assessment Method	TNM Stage	Histological Type (WHO)	Staging Criteria	Radiotherapy Technique
Chen 2014 (12)	Retrospective	2003-2007	54/100	MRI	RLN	I-II	AJCC 7th	IMRT
Guo 2006 (13)	Retrospective	1998-2000	80/76	CT/Color Doppler Ultrasound	N0	Not specified	FCSS 1992	2D-RT
Li 2005 (14)	Retrospective	1997-1998	88/90	Palpation	N0	I	FCSS 1992	2D-RT
Li 2013 (15)	RCT	2005-2012	153/148	MRI/CT	N0	II-III	AJCC 6th	2D-RT/IMRT
Ou 2012 (16)	Retrospective	2005-2009	89/30	MRI	RLN	I-II	AJCC 6th	2D-RT/3D-CRT/IMRT
Sun 2012 (17)	Retrospective	1989-2009	542/68	Palpation	N0	I-III	FCSS 1992	2D-RT/3D-CRT/IMRT
Tang 2009 (2)	Retrospective	2003-2004	37/101	MRI	N0	I-III	AJCC 6th	2D-RT/3D-CRT/IMRT
Xiao 2019 (18)	Retrospective	2009-2013	33/38	Not specified	N0/RLN	I	AJCC 8th	IMRT
Xie 2010 (19)	Retrospective	2002-2004	88/117	MRI/CT/PET-CT	N0	Not specified	UICC 6th	2D-RT
Zeng 2014 (9)	Retrospective	2003-2008	171/99	MRI/PET-CT	N0/RLN	II-III	AJCC 6th	IMRT
Zhang 2012 (20)	Retrospective	2005-2011	88/89	MRI/CT/Palpation	N0	II-III	FCSS 1992	2D-RT/3D-CRT

**Figure 2:**
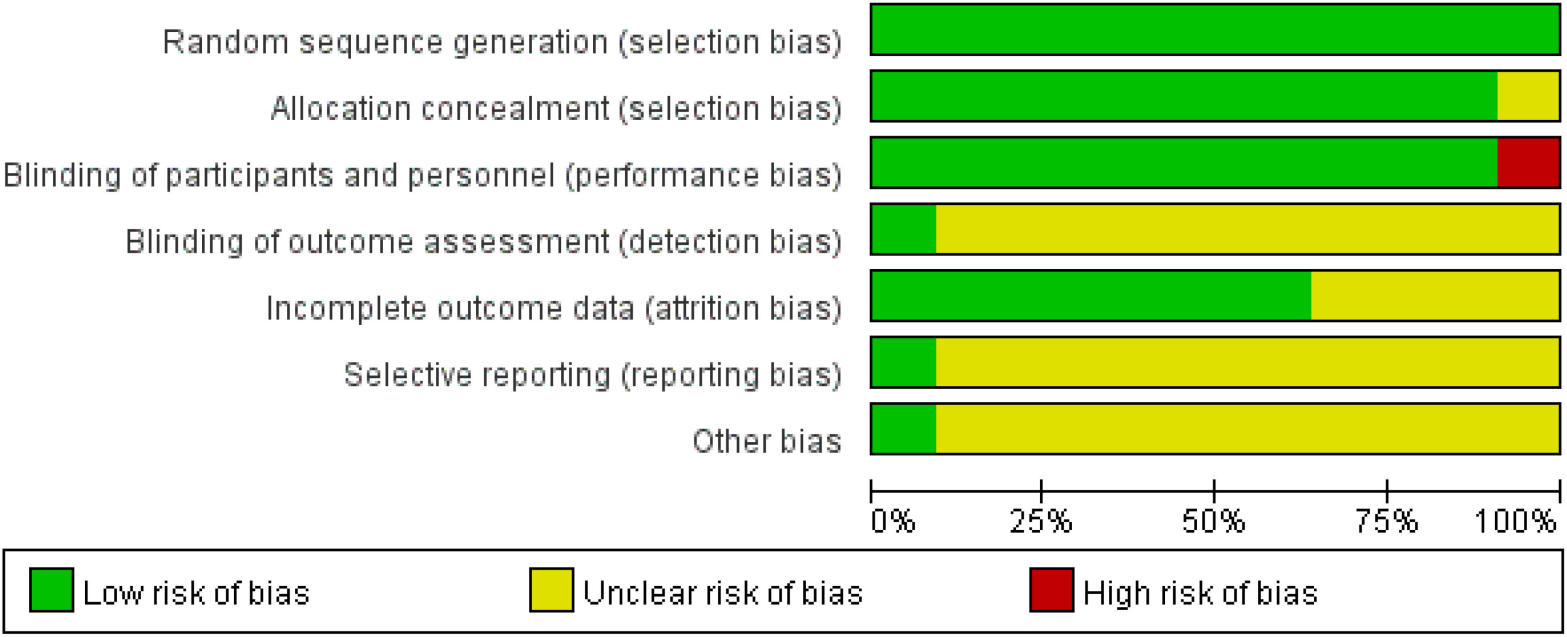
Risk of bias assessment for all included studies.

For the quality assessment of the 10 retrospective studies and the RCT, please refer to **Table 2** and **Table 3**. Among them, five retrospective studies and the RCT were classified as high quality, while the remaining five retrospective studies were considered to be of relatively lower quality. The risk of bias assessment for all included studies, based on the Cochrane Handbook guidelines, is illustrated in **Figure 2**.

**Table 2:** Quality assessment of the 10 retrospective studies.

Study or Subgroup	Study Population Selection				Intergroup Comparability	Exposure Measurement			Total Score
	Appropriateness of Case Determination	Representativeness of Cases	Selection of Controls	Definition of Controls	Consideration of comparability between cases and controls in design and statistical analysis	Determination of Exposure Factors	Using the same method to determine exposure factors for cases and controls	Nonresponse Rate	
Chen 2014 (12)	1	1	1	1	1	1	1	1	8
Guo 2006 (13)	1	1	1	1	1	1	0	0	6
Li 2005 (14)	1	1	1	1	1	1	0	0	6
Ou 2012 (16)	1	1	1	1	2	1	1	1	9
Sun 2012 (17)	1	1	1	1	1	1	1	1	8
Tang 2009 (2)	1	1	1	1	1	1	0	0	6
Xiao 2019 (18)	1	1	1	1	1	1	1	1	8
Xie 2010 (19)	1	1	1	1	1	1	1	1	8
Zeng 2014 (9)	1	1	1	1	1	1	1	0	7
Zhang 2012 (20)	1	1	1	1	1	1	1	0	7

**Table 3:** Literature quality assessment of 1 RCT.

Study or Subgroup	Random Sequence Generation	Allocation Concealment	Blinding	Withdrawals and Dropouts	Total Score
Li 2013 (15)	2	2	0	1	5

### 3.2 Survival outcomes

**Figure 3:**
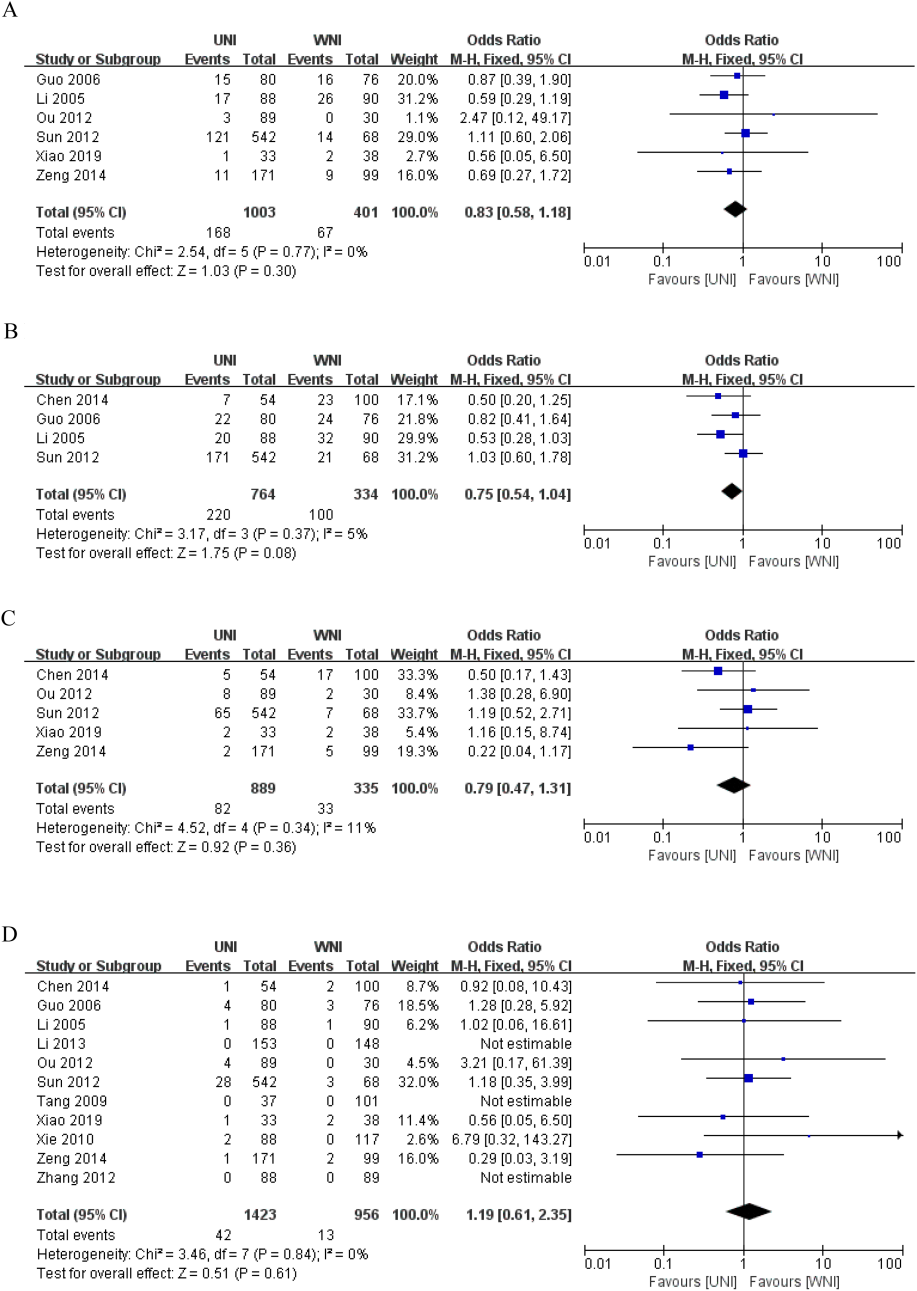
**(A)** Meta-analysis results of the UNI and WNI (5-year OS). **(B)** Meta-analysis results of the UNI and WNI (5-year DFS). **(C)** Meta-analysis results of the UNI and WNI (5-year DMFS). **(D)** Meta-analysis Results of UNI and WNI (Lymph Node Recurrence).

**Figure 3A** illustrates that, among 1404 patients from 6 studies (9, 13, 14, 16–18), there was no significant difference in 5-year overall survival (OS) between the UNI and WNI groups (OR=0.83, 95% CI=0.58-1.18; P=0.30). Additionally, there was no significant heterogeneity among these studies (P=0.77). **Figure 3B** presents pooled data from four studies (12–14, 17) comprising 1,098 patients, showing no statistically significant difference in 5-year disease-free survival (DFS) between the UNI and WNI groups (OR=0.75, 95% CI=0.54-1.04; P=0.08). Similarly, no significant heterogeneity was observed among the studies (P=0.37). **Figure 3C**, which includes pooled data from five studies (9, 12, 16–18) involving 1,224 patients, indicates no significant difference in 5-year distant metastasis-free survival (DMFS) between the UNI and WNI groups (OR=0.79, 95% CI=0.47-1.31; P=0.36). There was also no significant heterogeneity among these studies (P=0.34).

### 3.3 Lymph node recurrence

**Figure 4:**
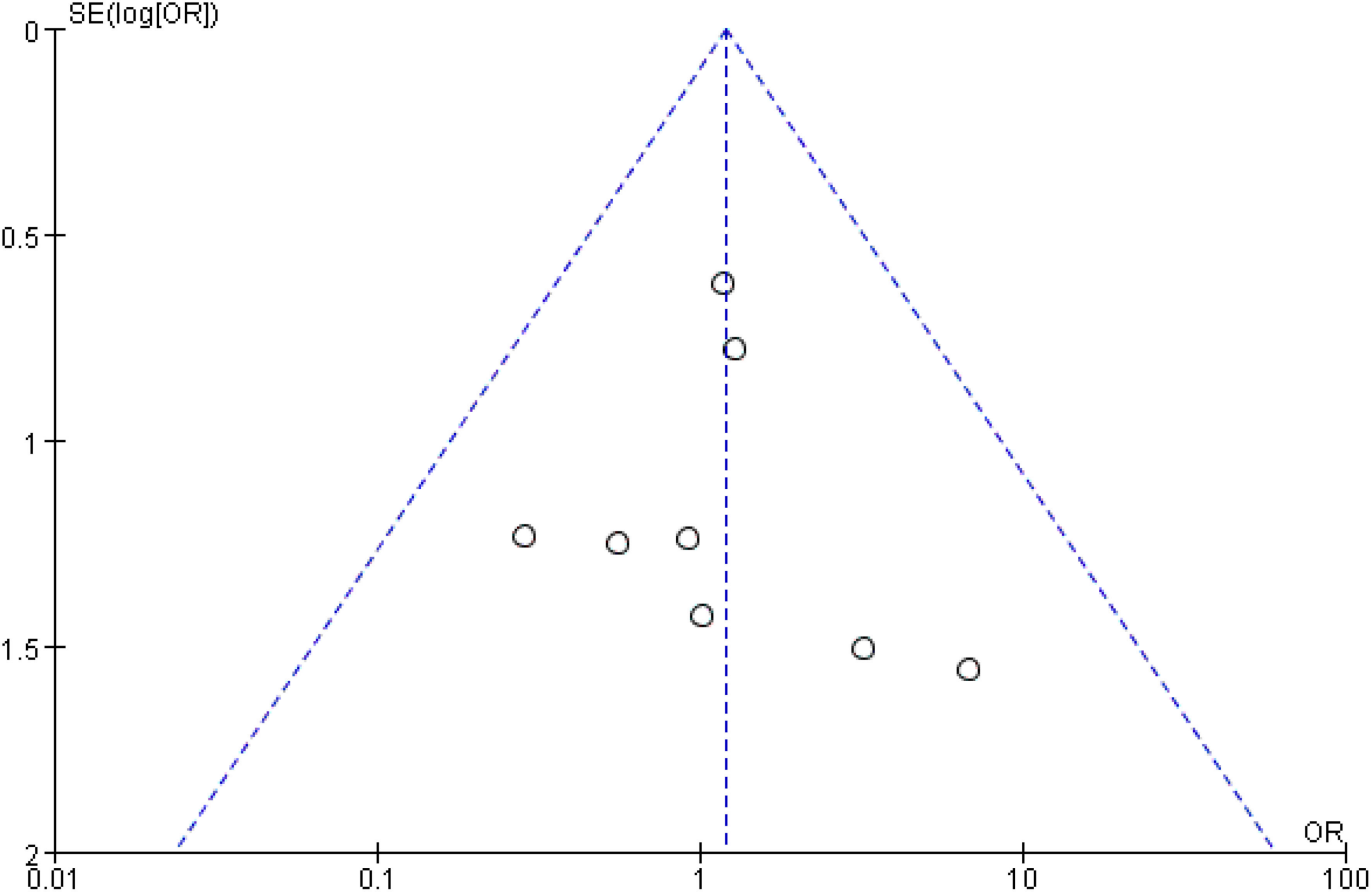
Funnel plot (lymph node recurrence).

As illustrated in **Figure 3D**, pooled data from 11 studies involving 2,379 patients demonstrated no significant difference in lymph node recurrence between the UNI and WNI groups (OR=1.19, 95% CI=0.61-2.35; P=0.61). Additionally, there was no significant heterogeneity among these studies (P=0.84). A funnel plot for lymph node recurrence, shown in **Figure 4**, indicates that all studies fell within the 95% CI range, suggesting no significant publication bias.

### 3.4 Subgroup analysis

**Figure 5:**
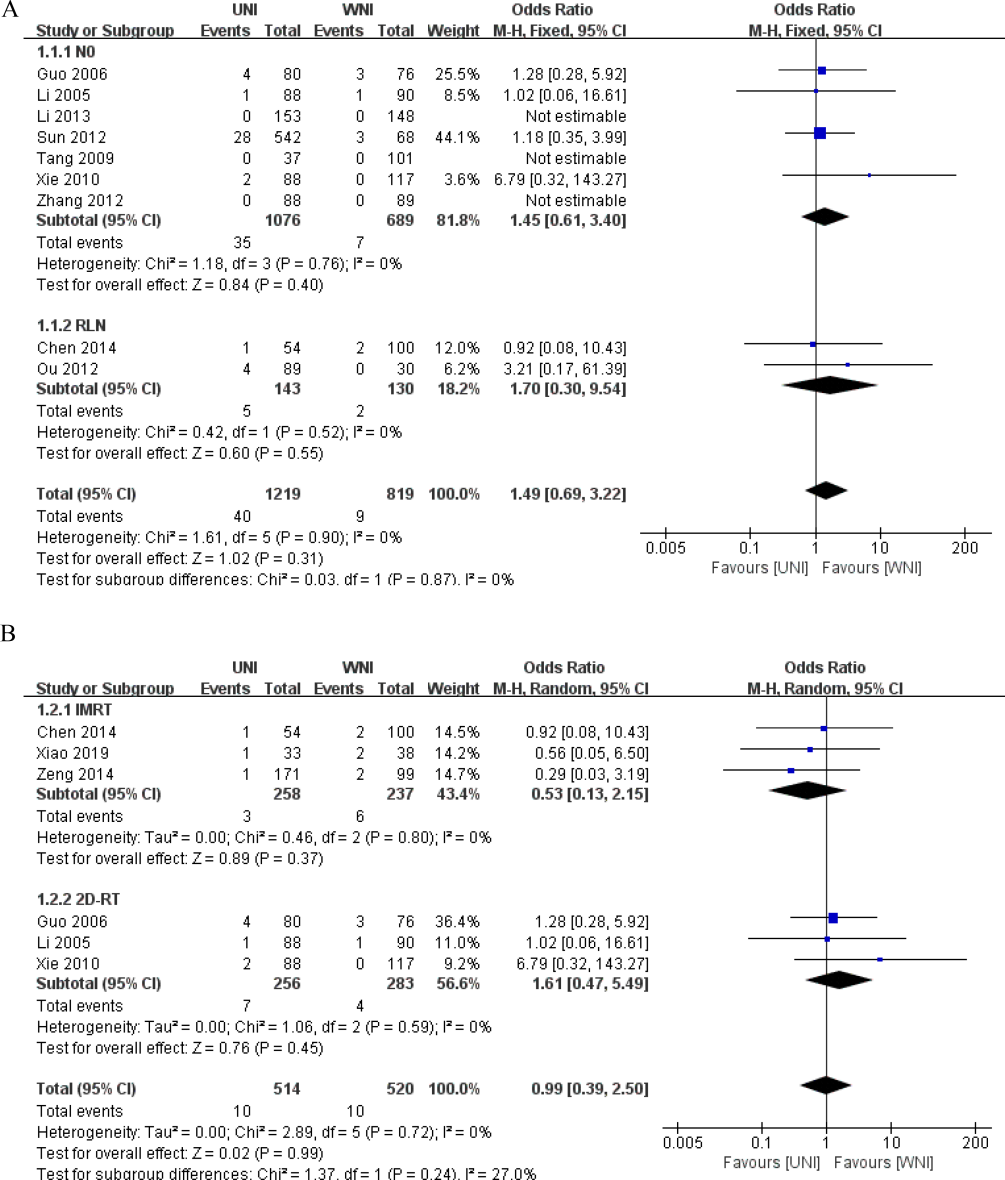
**(A)** Subgroup analysis of RLN metastasis status. **(B)** Subgroup analysis of radiotherapy techniques.

We conducted a subgroup analysis focusing on the metastatic status of the RLN and the radiotherapy techniques, using lymph node recurrence as the observational index. Seven studies reported lymph node metastatic status as N0 (2, 13–15, 17, 19, 20), two studies focused solely on RLN metastasis (12, 16), three studies utilized IMRT as the radiotherapy technique (9, 12, 18), and three studies employed 2D-RT (13, 14, 19). The subgroup analysis revealed no significant change in lymph node recurrence compared to the original meta-analysis results. Refer to **Figures 5A, B** for details.

### 3.5 Sensitivity analysis

**Figure 6:**
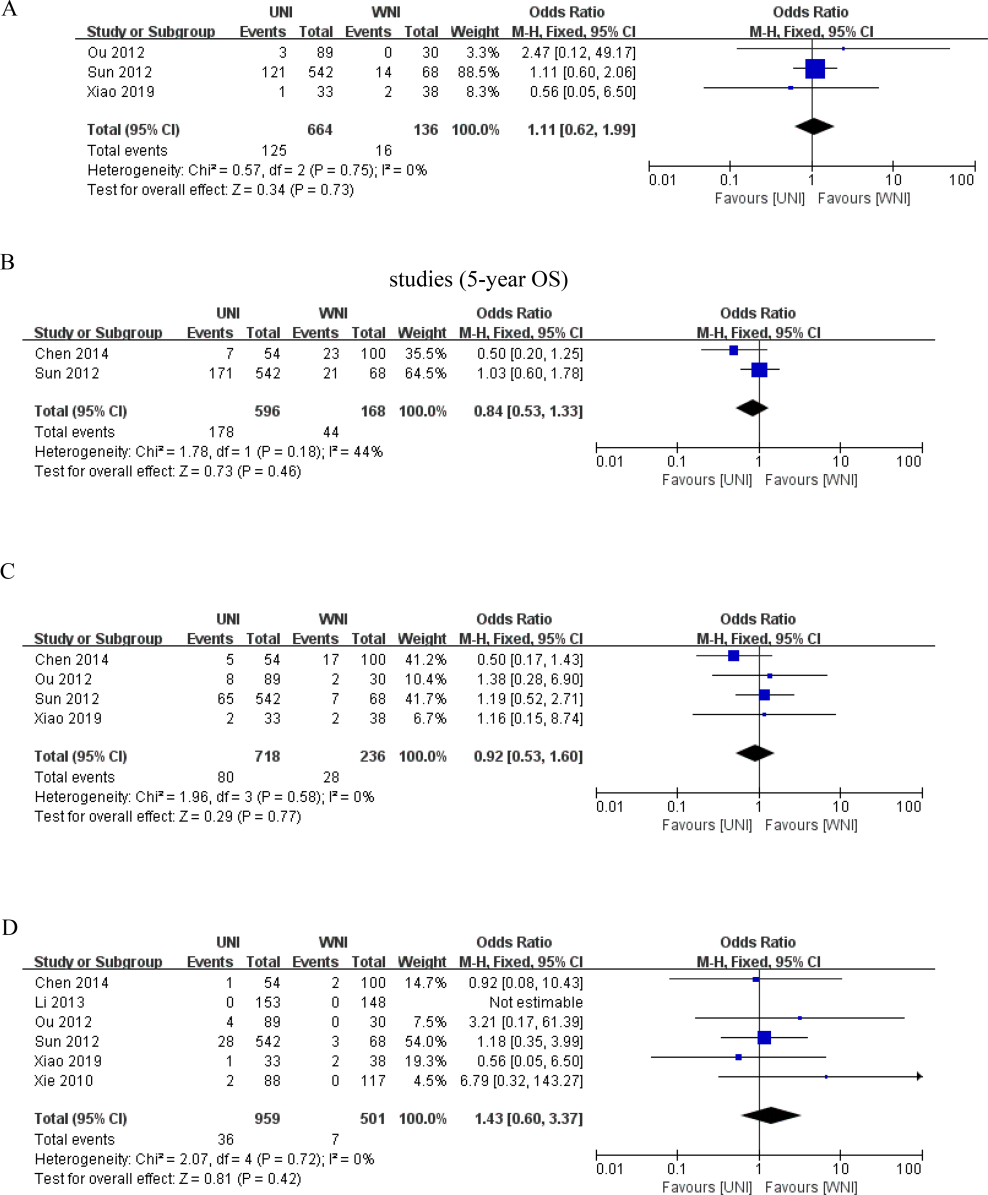
**(A)** Results of the meta-analysis of the UNI and WNI after excluding low-quality studies (5-year OS). **(B)** Results of the meta-analysis of the UNI and WNI after excluding low-quality studies (for 5-year DFS). **(C)** Meta-analysis results of the UNI and WNI after excluding low-quality studies (5-year DMFS). **(D)** Meta-analysis results of the UNI and WNI after excluding low-quality studies (lymph node recurrence).

We conducted sensitivity analyses by excluding five relatively low-quality studies to create forest plots for OS, DFS, DMFS, and lymph node recurrence. As illustrated in **Figures 6A–D**, the heterogeneity in 5-year OS, DFS, DMFS, and lymph node recurrence among the remaining studies was not significant (all *p*> 0.05), compared to the original meta-analysis. Thus, the findings of the original meta-analysis are deemed more reliable.

## 4 Discussion

Our meta-analysis of 11 studies revealed no significant differences in overall survival (OS), disease-free survival (DFS), distant metastasis-free survival (DMFS), or lymph node recurrence between the UNI and WNI groups for patients with cervical lymph node-negative nasopharyngeal carcinoma. These findings suggest that UNI is a safe and feasible treatment option for this patient population. Radiotherapy remains the cornerstone of treatment for nasopharyngeal cancer, and advancements in radiotherapy techniques have transitioned from traditional 2D-RT to 3D-CRT and, more recently, to IMRT (21). IMRT has significantly improved local regional control and survival rates in nasopharyngeal cancer patients by optimizing dose distribution, thereby reducing damage to surrounding normal tissues. According to Mao et al. (22), IMRT-based combination therapy for initially diagnosed, metastasis-free nasopharyngeal cancer achieves 5-year OS, DFS, DMFS, and nodal failure-free survival (NFS) rates of 82%, 75.1%, 82.6%, and 97%, respectively. Furthermore, a meta-analysis involving 3,570 participants demonstrated that patients treated with IMRT had significantly better 5-year OS (OR=1.51, 95% CI=1.23-1.87; P=0.0001) and local control (LC) of tumors (OR=1.94, 95% CI=1.53-2.46; P<0.00001) compared to those treated with 2D-RT or 3D-CRT. Additionally, IMRT significantly reduced radiation-induced toxic reactions (23).

Nonetheless, the damage to normal tissues caused by radiotherapy cannot be ignored. The currently accepted low-risk clinical target volume (CTV) for NPC typically includes the lower neck, regardless of lymph node status. However, this significantly increases the radiation dose received by the thyroid gland, carotid artery, lung apices, larynx, trachea, and soft tissues of the neck, leading to radiation-induced damage. It is estimated that approximately 40% of patients with nasopharyngeal cancer develop clinical or subclinical hypothyroidism after radiation therapy (24). Therefore, determining how to reduce radiation injury without compromising treatment efficacy is a primary focus in the field of radiotherapy. As previously mentioned, lymph node metastasis in nasopharyngeal carcinoma generally follows a sequential spread through the neck, with skip metastasis being rare. Tang et al. (2) used magnetic resonance imaging to retrospectively analyze the spread pattern of lymph node metastasis in nasopharyngeal carcinoma patients. Among the 786 patients studied, only 4 (0.5%) exhibited skip metastasis to the lymph nodes. Given this orderly spread pattern, is it feasible to change the target area of neck prophylactic irradiation from WNI to UNI for nasopharyngeal carcinoma patients with negative cervical lymph nodes?

Recent cutting-edge innovations in radiotherapy have primarily focused on narrowing the target area without compromising local control, thereby reducing radiotherapy-related toxicities and improving patient survival.

Jayson et al. (25) previously discussed the criteria for prophylactic neck irradiation in nasopharyngeal carcinoma with negative cervical lymph nodes or only RLN metastasis, and reached similar conclusions to this study. We have added subgroup analysis of RLN involvement and radiation therapy techniques based on it. RLN is located behind the nasopharynx and has a high metastasis rate due to its close anatomical location. About 70% of patients are already present at the initial diagnosis, so we should treat RLN differently from other cervical lymph nodes in terms of biological activity. Through subgroup analysis in this study, we found that nasopharyngeal carcinoma with only RLN metastasis still has a good prognosis and survival. Compared with the original meta-analysis, there was no significant change in lymph node recurrence. As for radiotherapy treatment techniques, from 2D-RT to 3D-RT, and then to IMRT, proton therapy, image-guided radiotherapy (IGRT), and adaptive radiotherapy (ART), nasopharyngeal carcinoma has become a disease that can be precisely cured. Through subgroup analysis, we found that the development of radiotherapy technology did not significantly affect the conclusions of this study. In the future, combining molecular imaging and biological targets, personalized radiotherapy based on tumor metabolic activity and different doses may become mainstream, achieving “personalized dose carving”, further improving efficacy and reducing toxicity.

Several factors should be considered when deciding between UNI and WNI for cervical lymph node-negative nasopharyngeal cancer: 1. Diagnostic methods for confirming cervical lymph node negativity. 2. Probability of skip metastasis in nasopharyngeal cancer. 3. Differences in survival outcomes between the two approaches. 4. Differences in radiotherapy-related toxicities.

This meta-analysis has several limitations: 1. RCTs accounted for a smaller proportion of the total studies, with a predominance of retrospective studies, which lowered the overall quality of the evidence. 2. Variations in clinical staging, lymph node metastasis assessment methods, and radiotherapy techniques across studies may have resulted in unbalanced baseline characteristics, potentially affecting the final results. 3. The included studies did not consistently report side effects and complications, precluding a meta-analysis of these outcomes. Given the lack of efficacy difference between UNI and WNI, side effects and complications warrant more attention. Future analyses should address this issue as more studies become available.

In conclusion, current evidence suggests that UNI is a safe and feasible option for nasopharyngeal carcinoma patients with negative cervical lymph nodes or only RLN metastases. UNI may reduce the radiation dose to normal tissues, potentially decreasing long-term adverse effects and improving quality of life. However, this meta-analysis should be considered preliminary due to the low overall quality of the included studies and the scarcity of RCTs. More RCTs and high-quality studies are needed to better assess the safety and feasibility of UNI.
